# Congenital Portosystemic Shunt Presenting As Hyperammonemia Following Fontan Operation

**DOI:** 10.1097/PG9.0000000000000282

**Published:** 2023-02-01

**Authors:** Kayla Morneault, April Mathews, Priya Sharma, Genie Beasley

**Affiliations:** From the * Department of Pediatrics, College of Medicine, University of Florida, Gainesville, FL; † Department of Radiology, College of Medicine, University of Florida, Gainesville, FL.

**Keywords:** Abernethy malformation, congenital heart defects, elevated central venous pressure

## Abstract

The Fontan operation allows survival for children with single ventricle congenital heart disease. In the acute postoperative period, perioperative insults and drastic changes in vascular pressures can potentially cause ischemic liver injury. We present a 3-year-old female with congenital heart disease presenting post-Fontan procedure complicated by altered mental status due to elevated ammonia levels. Etiology of the hyperammonemia was unknown and relatively controlled with medication. Further investigation, however, revealed a congenital portosystemic shunt. Congenital portosystemic shunts, more specifically Abernethy malformations, are rare conditions characterized as intrahepatic or extrahepatic, resulting in diversion of portal flow to systemic.

## INTRODUCTION

The Fontan operation increases survival in children with single ventricle congenital heart disease (CHD), creating passive flow of systemic venous return to the lungs, reducing intra-cardiac mixing, and improving oxygenation. It also increases systemic venous pressure and decreases preload, leading to venous congestion and decreased cardiac output, predisposing to liver injury, fibrosis, and eventual cirrhosis. In the acute postoperative period, perioperative insults and drastic changes in vascular pressures can cause ischemic liver injury (1).

We report a patient with acute symptomatic hyperammonemia and coagulopathy following a Fontan procedure. Evaluation revealed a portosystemic shunt due to circulatory changes induced by the procedure and symptom resolution following shunt occlusion.

## CASE REPORT

A 3-year-old female with double inlet left ventricle, transposition, and hypoplastic right ventricle, status post pulmonary artery banding and bidirectional Glenn procedure developed encephalopathy immediately following her Fontan procedure. She had hyperammonemia of 162 umol/L and an initial international normalized ratio of 2.3. Serum bilirubin and transaminase levels were normal, making ischemic liver injury unlikely. She was started on lactulose, which decreased the ammonia level to 100 umol/L. However, she developed elevation of aspartate transaminase and alanine transaminase levels to 45 and 58, respectively, and international normalized ratio worsened to 3.0, prompting treatment with vitamin K.

Abdominal Doppler ultrasound revealed sluggish flow in the portal vein but no hepatosplenomegaly. Computerized tomography angiography suggested a thrombus at the Fontan junction, which was refuted by cardiac catheterization. Transjugular free hepatic venous pressure was elevated at 14 mm Hg, but normal hepatic venous pressure gradient of 1 mm Hg disproved portal hypertension. Liver biopsy showed sinusoidal dilation and sinusoidal fibrosis attributed to pre-procedure cardiac disease but no cirrhosis or hepatitis. Metabolic, infectious, autoimmune, and genetic causes of liver disease were ruled out. Despite her presentation, she had no findings consistent with liver disease prior to the Fontan procedure.

Outpatient, her ammonia remained approximately 100 despite neomycin, rifaximin, lactulose, and low protein diet. Abdominal MRI, computed tomography, and ultrasound revealed no other etiology.

**FIGURE 1.**
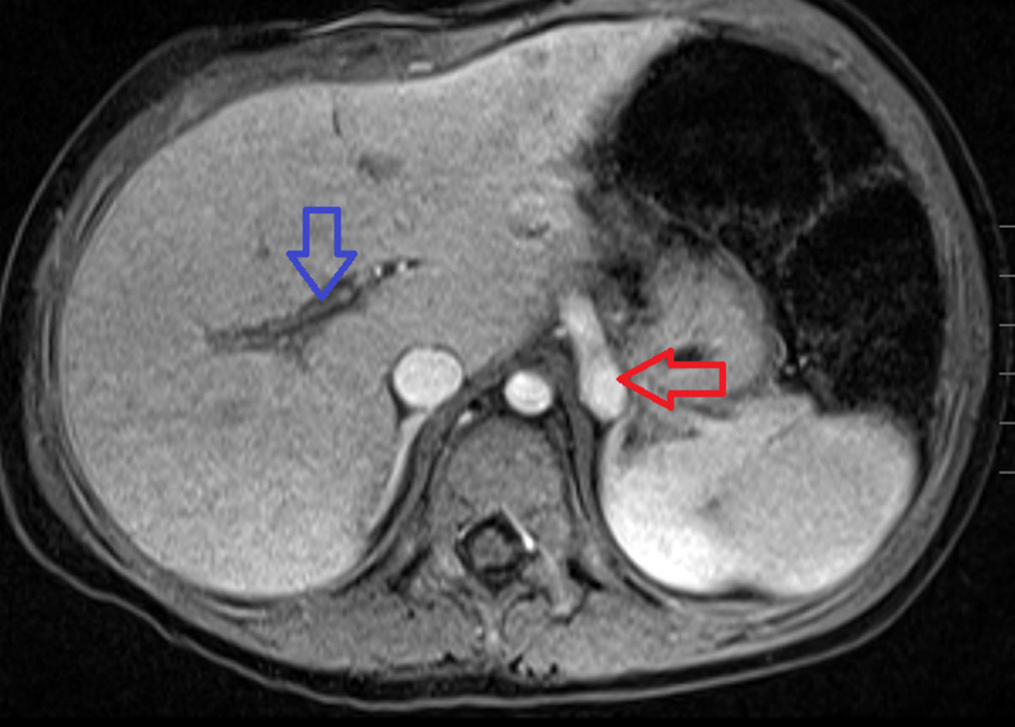
T1 post-contrast fat-saturated axial sequence demonstrating a large vessel (red arrow) in the left retroperitoneum above the level of the adrenal glands. This was later identified to represent a portosystemic shunt compatible with an Abernethy malformation. Note the hypoplastic portal vasculature (blue arrow). This is compatible with a type II Abernethy malformation.

**FIGURE 2.**
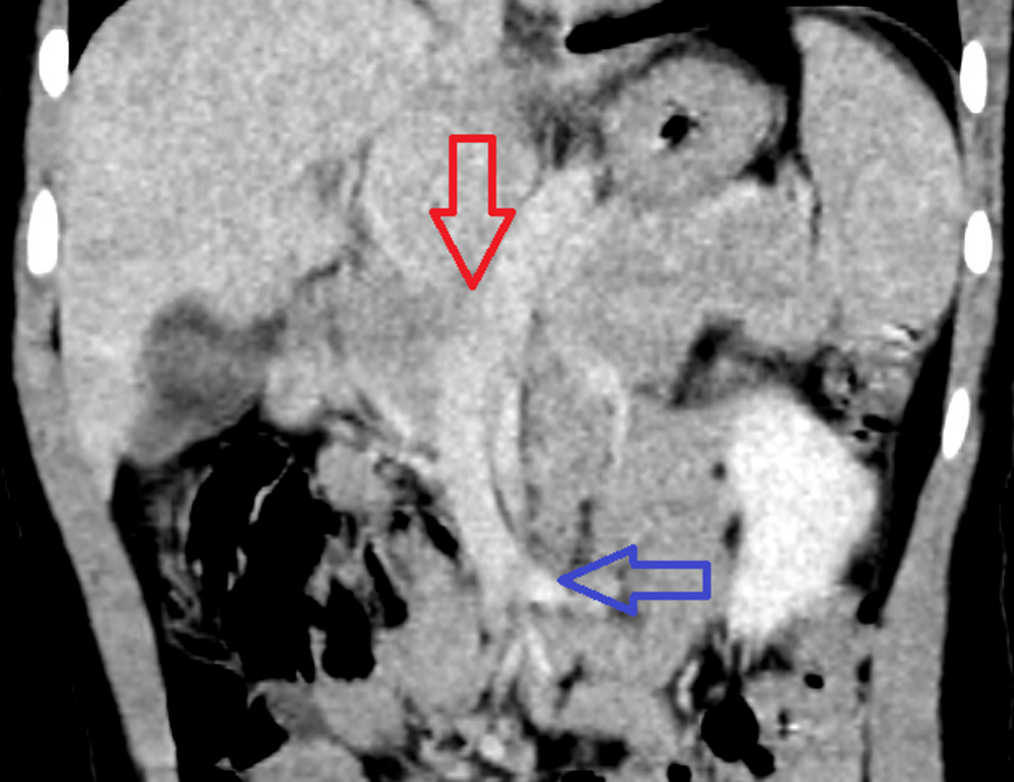
Coronal image of a CT of the abdomen and pelvis with IV contrast in the venous phase of imaging: There is a portosystemic shunt that opacifies during the during phase of imaging (red arrow). This vessel courses inferiorly and enters the confluence of the superior mesenteric vein (blue arrow). CT = computed tomography; IV = intravenous.

**FIGURE 3.**
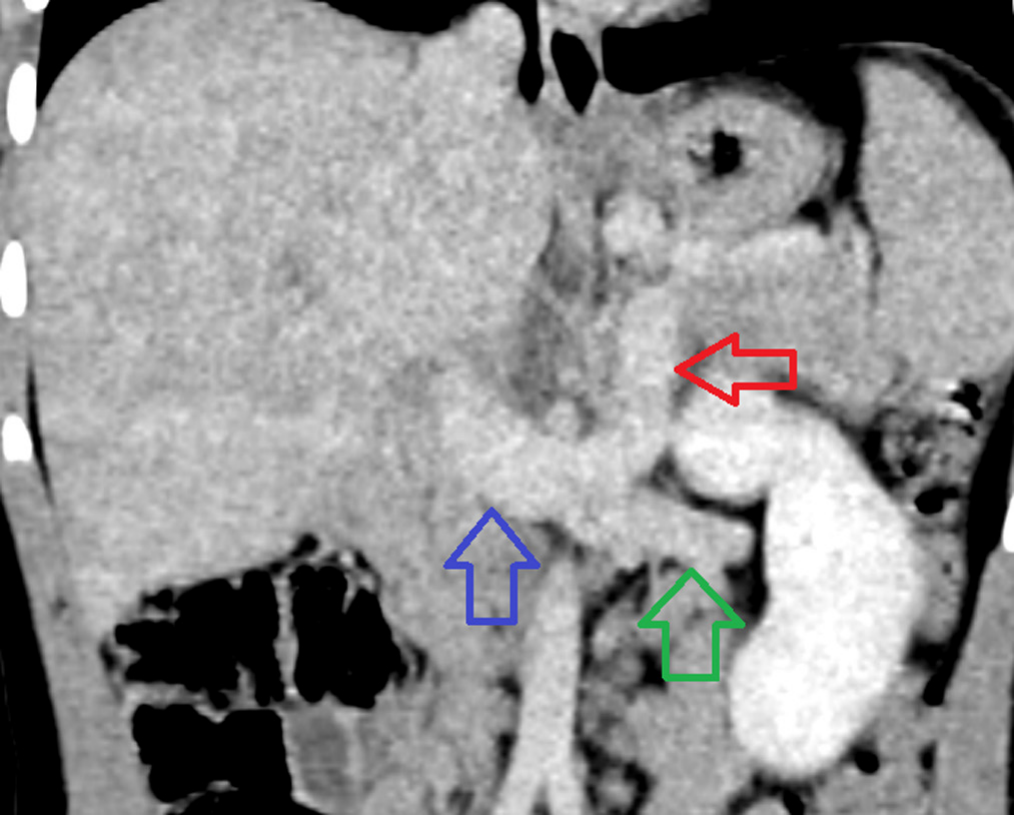
Coronal image of a CT of the abdomen and pelvis with IV contrast in the venous phase of imaging (red arrow). There is a portosystemic shunt that opacifies during the during phase of imaging. This vessel courses inferiorly and enters into the confluence of the left renal vein (green arrow) and IVC (blue arrow). CT = computed tomography; IV = intravenous; IVC = inferior vena cava.

Review by an outside consultant revealed a congenital portosystemic shunt and hypoplastic portal vasculature that in retrospect was visible on prior MRI and computed tomography imaging (Figs. 1–3). The shunt arose from the superior mesenteric vein and intersected the left renal vein, both draining into the inferior vena cava (IVC) consistent with Abernethy type II. Doppler ultrasound confirmed these findings. Angiography showed a diminutive but confluent intrahepatic portal venous system. Therefore, balloon angioplasty of portal veins was performed using a 2.5 mm by 20 mm Emerge balloon to optimize growth.

This was followed by partial shunt occlusion using a 10 mm Amplatzer vascular plug-I with a fenestrated device, which was a 3 mm by 18 mm Multilink vision stent. Occlusion of the residual shunt with a 14 mm Amplatzer vascular plug-II was required 8 months later, leading to resolution of hyperammonemia. Due to severely hypoplastic veins, a staged procedure was necessary to allow the portal veins time to grow between occlusions.

Six months later without further medical intervention, laboratory tests continued to demonstrate no concerns of liver injury or dysfunction with normal liver enzymes and ammonia levels.

## DISCUSSION

Hyperammonemia is defined as ammonia levels above 55 umol/L in children >1 month of age. Normally, ammonia is produced in the intestine, transported to the liver, and converted to urea via the urea cycle. Hyperammonemia results from enzyme defects, hepatocellular injury, diverted systemic circulation, or increased production. Thus, the importance lies in distinguishing the underlying cause, most commonly liver and biliary tract diseases or inborn errors of metabolism in the pediatric population (2).

In our patient, acute hyperammonemia prompted evaluation to rule out intrinsic hepatobiliary and metabolic causes. Elevated venous pressure was found, but without portal vein clot or signs of cirrhotic liver disease, a portosystemic shunt was suspected.

The incidence of congenital portosystemic shunts is estimated to be 1:30 000 births (3,4). The cause, whether congenital or acquired, is not well understood. Proposed explanations include genetic defects, congenital malformations, absence of the ductus venosus during fetal life, or hemangiomas of the liver (5). Others suggest the incomplete involution of the vitelline venous system or failure of the remodeling of anastomotic channels between vitelline and subcardinal veins (3).

These shunts can be classified as either intrahepatic or extrahepatic, with Abernethy malformations encompassing the extrahepatic category. They further subdivide into type 1a (superior mesenteric artery and splenic vein draining separately into the IVC), type 1b (superior mesenteric artery and splenic vein draining in a common trunk into the IVC), or type 2, which involves the portal flow partially diverted to systemic circulation with a preserved or hypoplastic main portal trunk connecting the IVC in a side-to-side anomaly (3). Our patient had both hypoplastic portal veins and a partial diversion making her a Type II malformation.

CHD, polysplenia, situs inversus, and genetic syndromes such as Downs, Rendu-Osler-Weber, and Noonans are examples of associated congenital abnormalities (3). The prevalence in CHD is unknown (6), but significant numbers of patients are suspected to have shunts. As discussed by Murray et al (7), of 61 patients with congenital extrahepatic portosystemic shunts, 31% also had CHD, suggesting an association. Similarly, DiPaola et al (8) found that CHD was an associated anomaly in 45% of the 11 patients in their report.

While post-Fontan patients are known to develop chronic liver changes leading to cirrhosis (4), liver disease uncommonly presents with acute hyperammonemia and a confounding congenital shunt as ours did. Our patient’s Fontan procedure led to a change in circulation. The IVC and SVC connecting to the pulmonary vasculature increased central venous pressure to allow for optimized blood flow into the heart, leading to portal congestion. The hypoplastic portal system exacerbated this by optimizing the increased blood flow through the Abernethy malformation as the pathway of least resistance in the vascular flow toward the IVC. Changes in flow caused the presentation of the malformation and hyperammonemia due to bypassing hepatic metabolization. Our patient’s presentation was atypical, lacked associated abnormalities, and therefore demanded consideration of less common etiologies of acute liver injury. Further studies regarding portosystemic shunts, their causes, and relationships to CHD are needed, especially as the population of those surviving Fontan operations continues to grow. Given the physiologic changes that can occur postoperatively, this case illustrates the raised index of suspicion needed in the differential diagnoses to consider vascular malformations and shunts in children with hyperammonemia.

## ACKNOWLEDGMENTS

Informed patient consent was obtained from the parents for publication of thedetails of this report.
